# Small peptide substrates and high resolution peptide gels for the analysis of site-specific protein phosphorylation and dephosphorylation

**DOI:** 10.14440/jbm.2017.199

**Published:** 2017-08-02

**Authors:** Laura Johnson Battle, Timothy C. Chambers

**Affiliations:** Department of Biochemistry and Molecular Biology, University of Arkansas for Medical Sciences, Little Rock, AR 72205, USA

**Keywords:** Cyclin-dependent kinase-1, Bcl-xL, caspase-9, peptide phosphorylation, peptide dephosphorylation, high resolution peptide gels

## Abstract

Protein phosphorylation and dephosphorylation reactions play key regulatory roles in many fundamental cellular processes. Due to the large number of kinases and phosphatases in the genome, the identification of the specific enzymes responsible for a given site in a given protein is immensely challenging. However, because protein kinases and phosphatases recognize local specificity determinants within proteins, it is possible to use small peptides to study the characteristics of site-specific phosphorylation. In addition, phosphorylation usually causes retardation in gel mobility, providing an opportunity to investigate peptide phosphorylation and dephosphorylation by monitoring migration on high resolution peptide gels. In this study, we demonstrate the utility of such a technique using small peptides corresponding to cyclin-dependent kinase-1 (Cdk1)/cyclin B1 sites in two important apoptotic regulatory proteins, Bcl-xL and caspase-9. We show that the mobility of the peptides is retarded following Cdk1-mediated phosphorylation, and that peptide dephosphorylation, catalyzed either by purified phosphatase or by crude cell extracts, is readily observable by increased peptide gel mobility. Furthermore, the procedure can be conducted without the use of radioactive adenosine triphosphate (ATP), and does not require any specialized reagents or apparatus. The method can be used to identify and characterize specific kinase and phosphatases responsible for phosphorylation and dephosphorylation of specific sites in any protein of interest.

## INTRODUCTION

Protein phosphorylation is a widespread mechanism for regulation of protein function [1-3]. Modification of proteins by phosphorylation is the primary mechanism for the transduction of intracellular signals, and is used as a means to regulate the activity of enzymes, protein-protein interactions, protein localization, and protein stability and degradation. Protein kinases phosphorylate specific serines, threonines or tyrosines in their substrates within the context of consensus sequences [4-6]. For example, cAMP-dependent protein kinase requires basic residues on the N-terminal side of the site to be phosphorylated, and many kinases, including mitogen-activated and cyclin-dependent protein kinases, require the presence of a proline residue on the C-terminal side of the site to be phosphorylated. Because the substrate specificity of protein kinases is principally determined by the local sequence around the phosphorylation site, small peptides are often effective as specific substrates and valuable as tools for kinase characterization. Indeed, peptide arrays serve as a powerful approach for kinase profiling [7].

Our interest has been in the role of cyclin-dependent kinase-1/cyclin B1 (Cdk1 henceforth) in the regulation on mitotic death [8-11]. Mitosis is a phase in the cell cycle where protein phosphorylation and dephosphorylation are prominently utilized because chromosomes are condensed, thus restricting the use of transcription (and translation) as a means for protein regulation [12-14]. We have shown that mitotic death is tightly associated with phosphorylation and inactivation of pro-survival Bcl-2 proteins including Bcl-2, Bcl-xL, and Mcl-1, with Cdk1 playing a key role [10,15]. Thus Cdk1, if inappropriately activated, can exert pro-death properties. Importantly, Cdk1 also exhibits pro-survival properties in mitotically arrested cells by phosphorylation and inactivation of caspase-9 [16]. Apoptosis is initiated when capase-9 becomes dephosphorylated, implicating the responsible phosphatase as a key downstream regulator and critical gatekeeper of mitotic death [17,18].

In order to facilitate investigation of Cdk1 substrates important in the regulation of mitotic death, we designed small peptides encompassing the major Cdk1-specific phosphorylation sites in Bcl-xL (Ser 62) [19] and caspase-9 (Thr 125) [16]. We show that these peptides undergo efficient phosphorylation by Cdk1 *in vitro*, and this is accompanied by a marked reduction in electrophoretic mobility when resolved using high resolution peptide gels. Furthermore, since peptide dephosphorylation converts it back to the faster migrating form, this offers a simple means for characterization of relevant phosphatases active in dephosphorylation of specific sites in these proteins. The methodology described is widely applicable and provides a convenient approach to characterize kinase and phosphatase activities responsible for site-specific phosphorylation and dephosphorylation.

## MATERIALS AND METHODS

### Materials

Purified active Cdk1/cyclin B1 was purchased from Millipore. Phosphocellulose P81 paper discs were purchased from Whatman. [γ-^32^P]ATP (250 µCi at 10 µCi/µl) was obtained from Perkin-Elmer. Precast 16.5% acrylamide Tris/Tricine gels, in an 18-well format, and peptide molecular mass standards were purchased from Bio-Rad. Lambda protein phosphatase was obtained from New England BioLabs. Other chemicals were obtained from Sigma.

### Peptides

Small peptides containing the major Cdk1 phosphorylation site in Bcl-xL (Ser 62) [19] or caspase-9 (Thr 125) [16] were synthesized at 95% purity by Genscript. The Bcl-xL derived peptide was designated FL-62 (for “flexible loop” region of Bcl-xL) [8] and corresponded to amino acid residues 58 to 66 with three arginines added to the C-terminus to facilitate binding to P81 phosphocellulose under acidic conditions (sequence HLADSPAVNRRR). The caspase-9 derived peptide was designated C-125 and represented amino acids 120-130 with three arginines added to the N-terminus to facilitate phosphocellulose binding (sequence RRRVLRPETPRPVD). Note that the choice of N- or C-terminal for attachment of the three arginines is arbitrary.

#### Peptide phosphorylation

FL-62 or C-125 peptides (50 µg) were incubated with or without 300 ng of active Cdk1/cyclin B1 at 30°C in kinase reaction mixtures of 100 µl containing 25 mM Tris-HCl [pH 7.5], 10 mM MgCl_2_, 5 mM dithiothreitol (DTT), 1 mM ATP, and 1 µCi [γ-^32^P]ATP. Aliquots containing 5 µg peptide were removed at intervals up to 8 h and reactions were stopped by the addition of 0.5 M ethylenediaminetetraacetic acid (EDTA) to 20 mM. For phosphorimage analysis, samples were boiled in sodium dodecyl sulfate (SDS) sample buffer, and a maximum of 10 µl was applied to 16.5% acrylamide Tris/Tricine high-resolution gels (referred to below as peptide-PAGE [polyacrylamide gel electrophoresis]). The gels were fixed in 5% glutaraldehyde overnight, washed extensively with distilled H_2_O, and exposed to a phosphorimager screen. To visualize peptides, gels were similarly fixed and washed with distilled H_2_O, stained with Coomassie Blue, and destained with 50% methanol, 10% acetic acid. Care should be taken not to destain too extensively as this causes peptide removal. To quantitatively determine phosphate incorporation into peptide, duplicate aliquots containing 5 µg peptide were removed at intervals, reactions were stopped by the addition of 0.5 M EDTA to 20 mM, the mixture was acidified by the addition of 90% v/v glacial acetic acid/H_2_O to 15%, and spotted onto 2-cm diameter P81 phosphocellulose filter discs. Filters were washed with 75 mM H_3_PO_4_ (4 × 500 ml), air dried, and ^32^P incorporation was measured by scintillation counting.

### Phosphatase treatment

FL-62 or C-125 peptides (150 µg in 300 µl) were phosphorylated as described above and the reaction mixture heated to 70°C for 10 min to inactivate Cdk1. Dephosphorylation by lambda phosphatase was carried out by adding 200 U lambda phosphatase for 2 h at 30°C. To test for the presence of cellular phosphatases active in peptide dephosphorylation, whole cell extracts were prepared from HeLa cells. Briefly, cells were harvested, washed, pelleted twice with cold phosphate-buffered saline (5 min at 500 × g), and resuspended in lysis buffer (25 mM HEPES [pH 7.5], 300 mM NaCl, 0.1% Triton X-100, 1.5 mM MgCl_2_, 0.2 mM EDTA, 0.5 mM DTT, EDTA-free complete protease inhibitor tablets [Roche], 20 µg/ml aprotinin, 50 µg/ml leupeptin, 10 µM pepstatin, and 1 mM phenylmethylsulfonyl fluoride. In some experiments, the lysis buffer was supplemented with phosphatase inhibitors (20 mM β-glycerophosphate, 1 mM sodium vanadate, 1 µM okadaic acid), and in other experiments, protease inhibitors were omitted. The suspension was incubated for 15 min on ice with occasional mixing, insoluble material was removed by centrifugation (20 min at 100000 × g), and the supernatant was retained as the whole-cell extract. Protein concentration was determined using Bio-Rad protein assay reagent. Aliquots containing 50 µg protein were incubated in a reaction mixture containing 25 mM Tris-HCl [pH 7.5], 10 mM MgCl_2_, 5 mM DTT, and 150 µg phosphorylated peptide, obtained as described above.

## RESULTS

### Phosphorylation of FL-62

**Figure 1.**
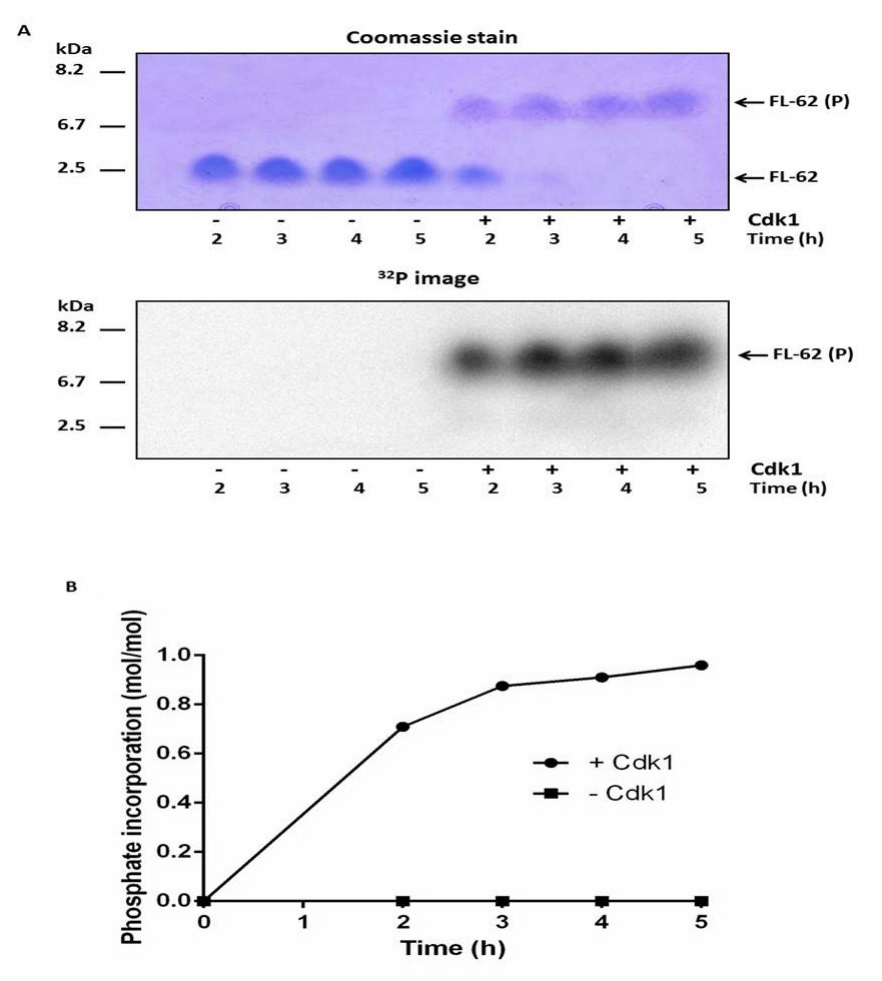
Phosphorylation of FL-62 peptide. **A.** FL-62 (50 µg) was incubated at 30°C under phosphorylation conditions in a reaction mixture of 0.1 ml containing 1 mM ATP and 20 µCi [γ-^32^P]ATP in the absence or presence of 300 ng active Cdk1/cyclin B1. At the indicated times, an aliquot (10 µl containing 5 µg FL-62) was removed and analyzed by peptide-PAGE, as described in the section of Materials and Methods. The gel was stained with Coomassie Blue, destained, and subjected to phosphorimager analysis. The migration positions of unphosphorylated and phosphorylated (P) FL-62 are indicated. The migrations positions of molecular mass standards are shown on the left. **B.** Aliquots (2 × 5 µl) of the same reaction were removed for the determination of phosphate incorporation, as described in the section of Materials and Methods. The results show the average of duplicate determinations and are representative of two additional independent experiments.

The small peptide FL-62, encompassing the major Cdk1 site in Bcl-xL, was incubated without or with active Cdk1/cyclin B1 under phosphorylation conditions using ^32^P-labeled ATP, as described in Methods and Materials. At timed intervals, aliquots were subjected to electrophoresis using a high resolution peptide gel, which was Coomassie stained and subjected to phosphor-image analysis. As shown in **Figure 1A**, the peptide migrated with an apparent molecular mass of about 2.5 kDa relative to molecular mass standards, as we described previously [8]. While the migration position of the peptide remained constant when Cdk1 was absent, in the presence of the kinase, peptide migration was markedly retarded. This appeared to be complete after 4–5 h of incubation. As shown in the corresponding phosphor-image, the shifted bands but not the unshifted bands were ^32^P-labeled, consistent with the mobility shift being due to phosphorylation. Aliquots of the reaction were also subjected to quantification of phosphate incorporation into peptides, as described in the section of Materials and Methods. **Figure 1B** presents a representative time-course of phosphorylation which was complete after 5 h, consistent with the gel-derived data, and reached a maximum of close to 1 mol of phosphate per mol of peptide (0.96 ± 0.06 mol/mol; mean ± SD, *n* = 3). Phosphate incorporation in the absence of Cdk1 was undetectable.

### Dephosphorylation of FL-62

**Figure 2.**
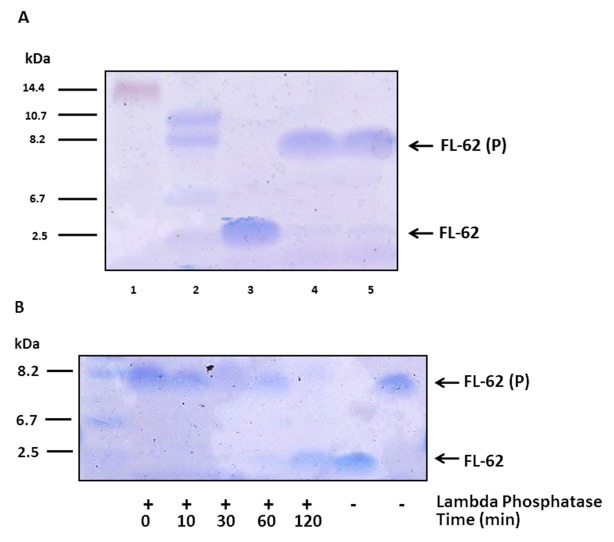
Dephosphorylation of FL-62. **A.** Inactivation of Cdk1 activity by heating. FL-62 peptide was incubated with Cdk1/cyclin B1 in a standard reaction mixture with non-radioactive ATP for 5 h at 30°C under the following conditions: lanes 1 and 2, molecular mass standards, as indicated on left; lane 3, mixture heated for 5 min at 70°C before the addition of ATP; lane 4, mixture was not heated prior to addition of ATP; lane 5, mixture was heated for 5 min at 70°C at the end of 5 h standard kinase reaction with ATP. Samples were resolved by peptide-PAGE and stained with Coomassie Blue. **B.** Dephosphorylation of phosphorylated FL-62. FL-62 was phosphorylated with active Cdk1 and ATP, and Cdk1 was then inactivated by heating, and phosphorylated peptide incubated with lambda phosphatase, as described under Materials and Methods. Aliquots containing 5 µg FL-62 were removed at the indicated intervals and subjected to peptide-PAGE and Coomassie Blue staining. In the right two lanes, unphosphorylated and phosphorylated FL-62 were analyzed as standards for comparison.

To determine if the phosphorylation-induced mobility shift was reversible after dephosphorylation, we sought to treat phosphorylated FL-62 with lambda phosphatase. However, the presence of active kinase in the initial reaction mixture represented a possible complicating factor. We considered isolating the peptide from the kinase reaction prior to treating with phosphatase, but due to the small amount present this proved inefficient and impractical. We therefore tested inactivating Cdk1 with heat after the kinase reaction, which would then allow the mixture containing phosphorylated peptide to be directly used as a source of phosphatase substrate. To demonstrate whether heat-inactivation was effective, we prepared a kinase reaction containing Cdk1 and FL-62 and heated it to 70°C for 5 min prior to the addition of ATP, and then carried out a normal incubation at 30°C for 5 h. As shown in **Figure 2A**, lane 3, the peptide failed to phosphorylate or shift in migration under these conditions. Without heat inactivation, the peptide became phosphorylated as shown by a shift in gel mobility (lane 4), and if the mixture was heated after the kinase reaction, the peptide remained intact and phosphorylated (lane 5). These results showed that heating was effective in Cdk1 inactivation without adverse effects on the peptide. Next, aliquots of kinase reactions containing phosphorylated FL-62 were heat-inactivated, incubated at 30°C with lambda phosphatase, and analyzed by high resolution gel electrophoresis. As shown in **Figure 2B**, we observed a time-dependent increase in FL-62 gel migration in the presence of phosphatase, with the peptide returning to the migration position of unphosphorylated FL-62 after 2 h. Thus electrophoresis of phosphorylated FL-62 using peptide gels provides a convenient method for examining phosphatases active in dephosphorylation.

### Phosphorylation of C-125

**Figure 3.**
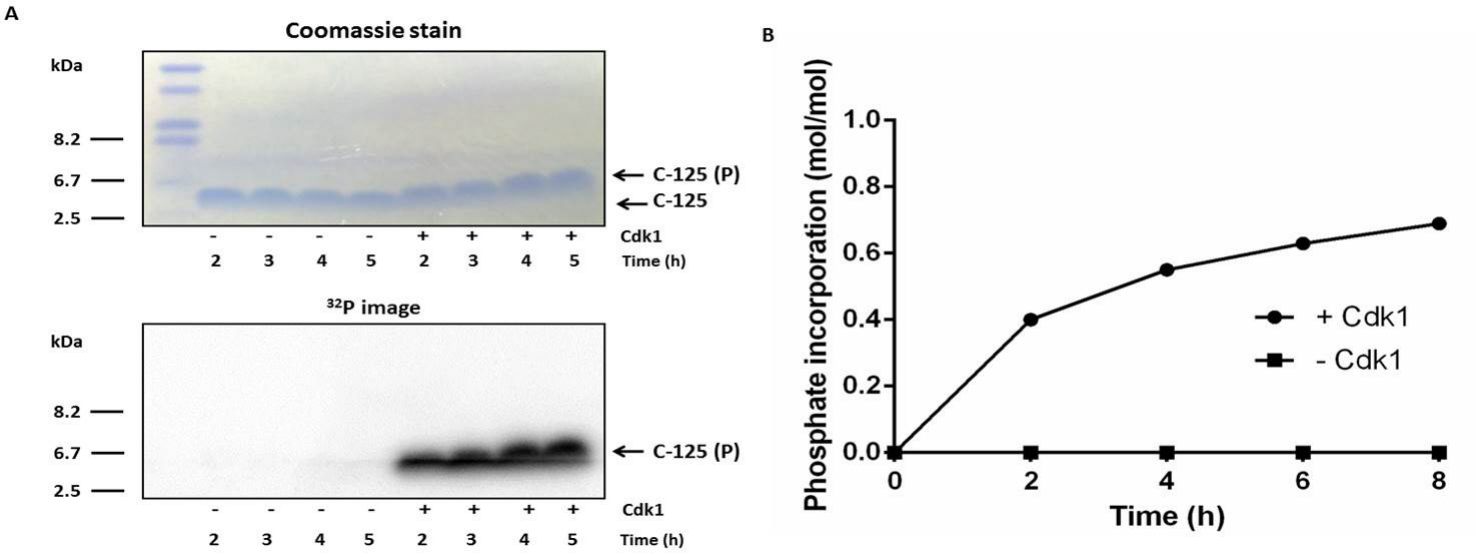
Phosphorylation of C-125 peptide. C-125 (50 µg) was incubated at 30°C under phosphorylation conditions with 1 mM ATP and 20 µCi [γ-^32^P]ATP in the absence or presence of 300 ng active Cdk1/cyclin B1. **A.** At the indicated times, an aliquot containing 5 µg was removed and analyzed by peptide-PAGE, as described in Materials and Methods. The gel was stained with Coomassie Blue, destained, and subjected to phosphorimager analysis. The migration positions of unphosphorylated and phosphorylated (P) C-125 are indicated. The migrations positions of molecular mass standards are shown on the left. **B.** Aliquots (2 × 5 µl) of the same reaction were removed for the determination of phosphate incorporation, as described in the section of Materials and Methods. The results show the average of duplicate determinations and are representative of two additional independent experiments.

We next studied phosphorylation of the small peptide C-125 encompassing the major Cdk1 site in caspase-9. C-125 was incubated without or with active Cdk1/cyclin B1 and subjected to electrophoresis using a high resolution peptide gel (**Fig. 3A**). The unphosphorylated peptide migrated with an apparent molecular weight of about 4 kDa and underwent a progressive retardation in mobility after incubation with Cdk1 under phosphorylation conditions. The mobility shift, though readily evident, was not as striking as that observed for FL-62. As shown in the corresponding phosphor-image (**Fig. 3A**), the shifted bands were ^32^P-labeled, confirming that the peptide underwent phosphorylation. Quantification of phosphate incorporation from a representative experiment is shown in **Figure 3B.** A stoichiometry of 0.74 ± 0.07 (mean ± SD) mol phosphate per mol of peptide was derived from three determinations after 8 h incubation.

### Dephosphorylation of C-125

**Figure 4.**
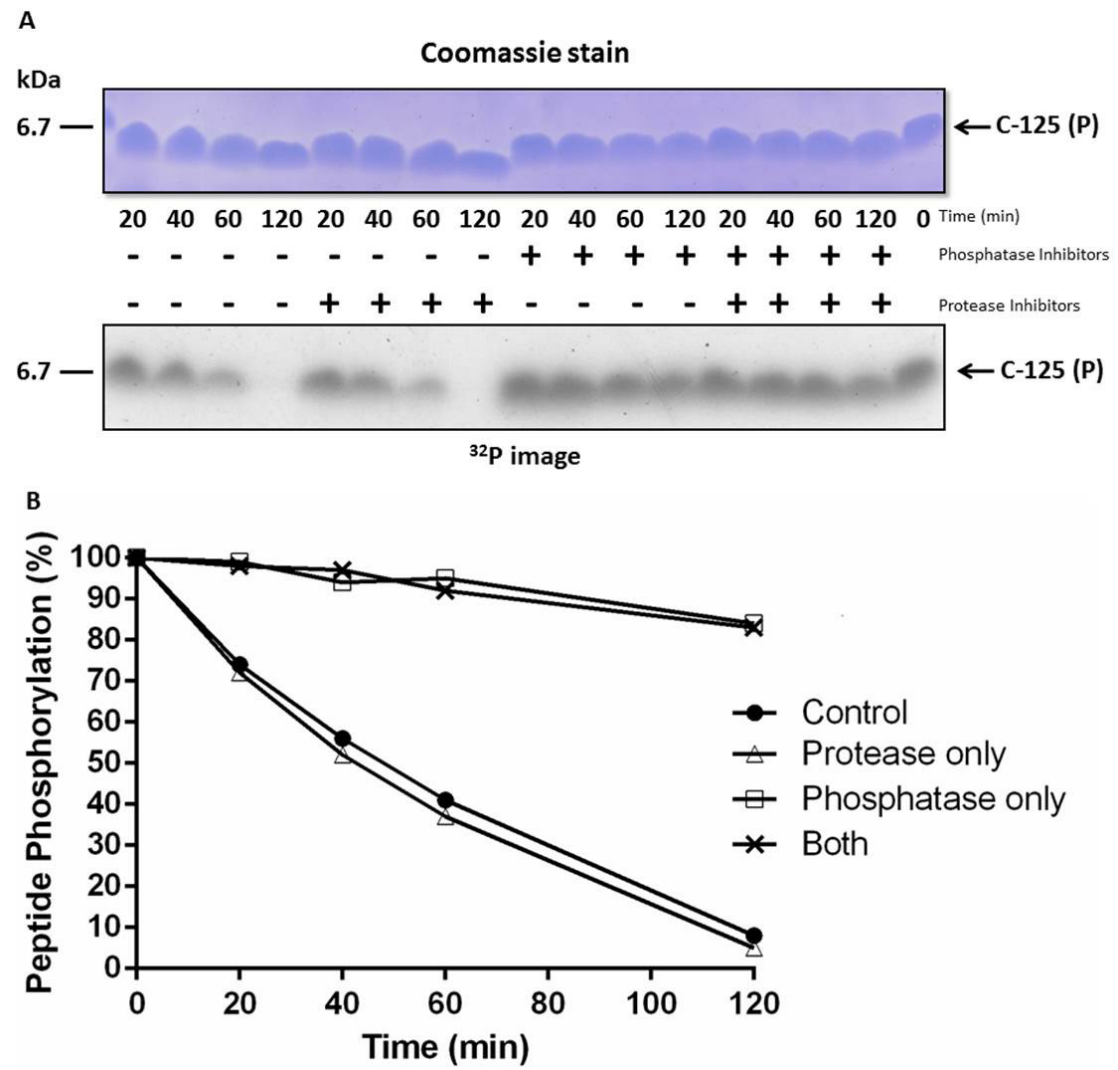
Dephosphorylation of C-125 peptide. C-125 was phosphorylated with active Cdk1 and ATP, and Cdk1 was then inactivated by heating, and phosphorylated peptide incubated with HeLa cell extracts, as described under Materials and Methods. The cell extracts were prepared in the absence or presence of protease or phosphatase inhibitors, as indicated. **A.** Aliquots containing 5 µg C-125 were removed at the indicated intervals and subjected to peptide-PAGE and Coomassie Blue staining and phosphorimage analysis. In the far right lane, phosphorylated C-125 was analyzed as a standard for comparison. **B.** Aliquots (2 × 5 µl) of the same reactions were removed for the determination of phosphate incorporation, as described in Materials and Methods. The results show the average of duplicate determinations and are representative of one additional independent experiment.

In order to study C-125 dephosphorylation, we incubated ^32^P-phosphorylated C-125 with whole cell lysates from HeLa cells, as described in the section of Materials and Methods, and under four different conditions: (1) in the absence of protease and phosphatase inhibitors; (2) in the presence of protease inhibitors but absence of phosphatase inhibitors; (3) in the presence of phosphatase inhibitors but absence of protease inhibitors; or (4) in the presence of both. As shown in **Figure 4A**, lanes 1–4, in the absence of both types of inhibitors, phosphorylated C-125 became progressively dephosphorylated in a time-dependent manner, as shown both by faster migration of the stained peptide, and by loss of ^32^P-content observed by phosphorimager analysis. Essentially identical results were observed when phosphorylated C-125 was incubated in the presence of protease inhibitors but absence of phosphatase inhibitors (**Fig. 4A**, lanes 5–8). In contrast, in the presence of phosphatase inhibitors and irrespective of the presence or absence of protease inhibitors, C-125 phosphorylation was maintained throughout the course of incubation, as indicated by preservation of gel mobility and by retention of ^32^P-content (**Fig. 4A**, lanes 9–16). These results also show that there is no overt degradation of the peptide during the course of incubation and thus protease inhibitors are dispensable. Aliquots of the same samples were subjected to quantitative determination of ^32^P incorporation into peptides, as described in the section of Materials and Methods (**Fig. 4B**). These results graphically illustrate the ability of phosphatase inhibitors to maintain C-125 in a phosphorylated state, with preservation of over 80% of the ^32^P-phosphate originally present in the peptide, whereas in the absence of phosphatase inhibitors, essentially complete dephosphorylation occurred.

## DISCUSSION

Protein phosphorylation and dephosphorylation is a widespread means for regulation of protein function [1-3]. Because many proteins are phosphorylated on multiple sites by multiple kinases [20], the use of small peptide substrates containing individual consensus sequences has facilitated and simplified protein phosphorylation research. One example is our previous work focused on identification of the kinase which catalyzes mitotic arrest-induced Bcl-xL phosphorylation. The use of the small peptide FL-62, described in more depth here, was instrumental in identification of Cdk1/cyclin B1 as the key responsible kinase, since full length Bcl-xL proved to be a non-selective substrate phosphorylated by many kinases in the purified extracts we employed for the study [8]. In this paper, we describe studies with two peptides containing Cdk1 sites, one derived from Bcl-xL and one from caspase-9, together with analysis using high resolution Tris-tricine peptide gels. The results illustrate their utility as substrates for the characterization of kinase and phosphatases catalyzing their phosphorylation and dephosphorylation, respectively. The stoichiometry of phosphorylation of each peptide, of close to 1 mol phosphate/mol peptide for FL-62 and about 0.7 mol/mol for C-125, is consistent with phosphorylation of the single phosphoacceptor site present in each. Both peptides underwent gel retardation upon phosphorylation, and it is expected that similarly sized peptides containing phosphoacceptor sites derived from other proteins will exhibit this same property. The gel mobility of FL-62 was much more markedly retarded after phosphorylation than was C-125. While the basis for this observation is not clear, phosphorylation might promote the formation of stable FL-62 dimers or trimers for example, or could reduce SDS binding to a greater degree for FL-62 than C-125. An important advantage of this approach is that peptide phosphorylation can be readily monitored using non-radioactive ATP. However, this does depend on the peptide in question undergoing an observable gel mobility shift after phosphorylation, Optimization of electrophoresis conditions and the use of gradient acrylamide gels might be necessary to maximize mobility shift. In addition, this approach lends itself well to testing different kinases for their ability to phosphorylate specific sites. For example, Ser 62 in Bcl-xL has been reported to be phosphorylated by c-Jun N-terminal kinase, based on a strategy where putative sites in Bcl-xL were mutated [21]. The availability of FL-62 provides an opportunity to determine whether JNK or other kinases have the ability to phosphorylate this site, which we characterized as being phosphorylated by Cdk1 [8,19].

Since many chemotherapeutic drugs, and especially microtubule targeting agents, promote mitotic arrest and subsequent cell death, there is considerable interest in the molecular mechanism coupling these two events [22,23]. One intriguing mechanism involves regulation of caspase-9 which has been reported to be maintained in an inactive state during mitotic arrest by Cdk1-mediated phosphorylation of Thr 125 [16]. Apoptosis is hypothesized to occur when caspase-9 becomes dephosphorylated resulting in caspase-9 activation. We have also presented evidence that when mitotic death is triggered in HeLa cells, it occurs concomitantly with widespread phosphatase activation [24]. Therefore, there is much interest in characterizing the phosphatase responsible, as it may be a key link between mitotic arrest and mitotic death. Cdk1-phosphorylated C-125, described here, represents a specific and convenient substrate with which to characterize this key phosphatase, and we have shown the feasibility of this approach by demonstrating C-125 phosphatase activity in HeLa cell extracts (**Fig. 4**).

In conclusion, we have illustrated the potential of small peptides containing phosphorylation sites coupled with high resolution peptide PAGE for the characterization of site-specific phosphorylation and dephosphorylation. The method is widely applicable, does not require radioactive ATP, and provides a convenient means to identify kinases and phosphatases responsible for phosphorylation and dephosphorylation of specific residues in any protein of interest.
